# Perceived pros and cons of smoking and quitting in hard-core smokers: a focus group study

**DOI:** 10.1186/1471-2458-14-175

**Published:** 2014-02-18

**Authors:** Jeroen Bommelé, Tim M Schoenmakers, Marloes Kleinjan, Barbara van Straaten, Elske Wits, Michelle Snelleman, Dike van de Mheen

**Affiliations:** 1 IVO Addiction Research Institute, Heemraadssingel 194, 3021 DM Rotterdam, The Netherlands; 2 Erasmus Medical Centre, Dr. Molewaterplein 50, 3015 CE Rotterdam, The Netherlands; 3 Behavioural Science Institute, Radboud University Nijmegen, Montessorilaan 3, 6525 HR Nijmegen, The Netherlands; 4 Department of Health Promotion, Maastricht University, P.O. Box 6166200 MD Maastricht, The Netherlands

**Keywords:** Hard-core smokers, Pros and cons, Smoking, Quitting, Focus groups, Netherlands

## Abstract

**Background:**

In the last decade, so-called hard-core smokers have received increasing interest in research literature. For smokers in general, the study of perceived costs and benefits (or ‘pros and cons’) of smoking and quitting is of particular importance in predicting motivation to quit and actual quitting attempts. Therefore, this study aims to gain insight into the perceived pros and cons of smoking and quitting in hard-core smokers.

**Methods:**

We conducted 11 focus group interviews among current hard-core smokers (n = 32) and former hard-core smokers (n = 31) in the Netherlands. Subsequently, each participant listed his or her main pros and cons in a questionnaire. We used a structural procedure to analyse the data obtained from the group interviews and from the questionnaires.

**Results:**

Using the qualitative data of both the questionnaires and the transcripts, the perceived pros and cons of smoking and smoking cessation were grouped into 6 main categories: Finance, Health, Intrapersonal Processes, Social Environment, Physical Environment and Food and Weight.

**Conclusions:**

Although the perceived pros and cons of smoking in hard-core smokers largely mirror the perceived pros and cons of quitting, there are some major differences with respect to weight, social integration, health of children and stress reduction, that should be taken into account in clinical settings and when developing interventions. Based on these findings we propose the ‘Distorted Mirror Hypothesis’.

## Background

In the last decade, so-called hard-core smokers have received increasing interest in research literature. According to some, their significance within the population of smokers will increase over the coming years [1]. Although many different definitions exist, most agree that smokers are considered ‘hard-core’, when they have a high level of nicotine dependence, have smoked for a considerable number of years and, most importantly, show little to no intention to quit [2].

According to the *hardening hypothesis*, current anti-smoking policies are more likely to affect smokers who are less dependent on tobacco than those who are more dependent [3]. Therefore, light smokers (i.e., smokers who smoke less cigarettes per day, who are more willing to quit, or who experience less symptoms of nicotine dependence) are more likely to cease smoking than hard-core smokers. In other words, current policies and interventions tend to make light smokers quit, leaving a larger portion of hard-core smokers in the total population of smokers [4]. Although the hardening hypothesis has faced mixed evidence [5], research has shown that hard-core smokers are less likely to be affected by tobacco control measures [6]. This emphasises the importance of developing interventions targeting hard-core smokers.

For smokers in general, the study of perceived costs and benefits (or ‘pros and cons’) of smoking is particularly important in predicting motivation to quit and actual quitting attempts [7-9]. Many theories, like the Health Belief Model [10], the Theory of Planned Behaviour [11], the Transtheoretical Model [12], and the Social Cognitive Theory [13] acknowledge the influence of perceived pros and cons in the process of behavioural change. Evidence suggests that hard-core smokers differ from non-hard-core smokers in their perceived pros and cons of smoking and quitting. For example, hard-core smokers are less likely to consider smoking as a possible cause of health damage for themselves [14] and they are also less likely to acknowledge the possible adverse health effects of second hand smoking [6]. However, until now, relatively little is known about the perceived pros and cons of smoking and smoking cessation in the specific subgroup of hard-core smokers. Knowledge on the attitudes of hard-core smokers towards smoking and smoking cessation may help to develop interventions specifically targeting this group.

Although research on smoking cessation in general population smokers has yielded substantial knowledge about the perceived pros and cons of smoking, there is a lack of central focus. Some studies only investigate the perceived pros and cons of smoking [15-17], while others only target the perceived pros and cons of smoking *cessation* (or ‘quitting’) [18,19]. Some attempted to combine both concepts [20], but did not explicitly investigate the four different perspectives involved (i.e., pros of smoking, cons of smoking, pros of quitting, and cons of quitting).

We argue that it is important to assess all four perspectives explicitly to obtain the most comprehensive view on attitudes towards smoking and quitting. For example, smokers may see many pros and few cons of quitting but may keep on smoking for just one perceived proof smoking (e.g. it helps them to relax). Moreover, the perceived pros and cons of smoking do not necessarily mirror the perceived pros and cons of quitting. Smokers may, for instance, smoke to feel socially accepted by friends, but may not necessarily think that quitting would make them less accepted by friends. Investigating all four perspectives may reveal contradictory beliefs that (hard-core) smokers have towards smoking and quitting. In this study we therefore investigate all four perspectives in hard-core smokers.

Former hard-core smokers have successfully gone through the process of quitting and they might provide additional insights into the balance of motives to stop or to continue smoking. Current hard-core smokers, who have not yet permanently quit, might lack the experience to identify the crucial pros or cons that might tip the balance of motives from smoking continuation towards smoking cessation.

In summary, in the present study we investigated the perceived pros and cons of smoking and quitting among hard-core smokers by conducting a focus group study among low and high SES current hard-core smokers, and among low and high SES former hard-core smokers. The aim of the current study was to gain insight into the perceived pros and cons of both smoking and smoking cessation in hard-core smokers.

## Methods

### Participants

Participants were recruited via an online survey sample (Survey Sampling International, SSI). Over 5000 Dutch panel members were invited to fill out a small screener designed to identify eligible participants. Participants were eligible if they were current or former hard-core smokers.

Previous studies identify three basic characteristics of hard-core smokers: relative high tobacco consumption, little intention to quit, and resilience to societal pressures as indicated by a relatively long smoking history. We translated these into six criteria for our screener. Smokers were defined as hard-core if they a) smoked every day [6,21], b) smoked on average 15 cigarettes or more a day [6,21], c) had not attempted to quit smoking in the past year [6,14,21,22], d) were not planning to quit within 6 months [6,14,21,22], e) had been smoking at least 15 years in their lifetime, and f) were 35 years or older. As for the last criterion, we selected these older smokers, because smoking-related pros and cons tend to differ between younger and older smokers [23]. They have surpassed young adulthood and have reached a stable smoking habit with commensurable smoking-related cognitions.

Former hard-core smokers were also aged 35 or older and had been smoking at least 15 years in their lifetime. All participants had been smoking more than 15 cigarettes daily at one point in their life. All former hard-core smokers had stopped smoking for at least one year at the time of the interviews.

We identified about 1350 current and about 900 former hard-core smokers, of which 314 current and 132 former hard-core smokers were interested in attending a focus group interview. After exclusion of participants who were unable to attend due to time and/or geographical limitations (all focus groups were conducted in the same two cities, restricting our sample to those participants who lived nearby or were willing to travel far), 31 former and 32 current hard-core smokers participated in our focus group study.

All participants were aged 35–79 (M = 54.7, SD = 7.6) and groups sizes were 2–10. Table 1 presents the background characteristics of the current and former smokers. We used t-tests to analyse differences between the two groups. At the start of the interview, all participants introduced themselves and all but two former smokers indicated the number of years they had quit. This ranged between 1.5 and 40 years (M = 10.0, SD = 8.1). Participants received compensation for their travel expenditures and an additional 45 euros for their participation.

**Table 1:** Sample characteristics

	**Current smokers (n = 32)**	**Former smokers (n = 31)**
Demographics		
Female, N (%)	11 (34.4%)	11 (35.5%)
Age (SD)^a^	52.7 (7.0)	56.8 (7.6)
Years smoked in life (SD)^a, b^	36.5 (8.2)	31.0 (9.9)
Socioeconomic status^c^		
Low	18 (56.3%)	18 (58.1%)
High	14 (43.8%)	13 (41.9%)
Intention to quit		
Within 1 year	4 (12.5%)	
Within 5 years	4 (12.5%)	
Not quitting, but smoking less	12 (37.5%)	
Not quitting and not smoking less	12 (37.5%)	
Nicotine dependence		
FTND (SD)^d^	6.13 (1.5)	5.97 (1.8)
Cigarettes per day (SD)^d^	26.7 (8.1)	32.7 (16.9)

^a^Significant difference between current and former hard-core smokers (p < .05).

^b^For all participants a minimum of 15 years was required. ^c^Socioeconomic status was measured as the highest completed level of education. ^d^Former smokers filled out the Fagerström Test for Nicotine Dependence (FTND) for the period “they smoked the most”.

### Procedure

Using the standardized procedures of Goldman and Schmaltz [24], we conducted 11 focus group interviews among current hard-core smokers (n = 32) and former hard-core smokers (n = 31) in the Netherlands. Focus group research is a research method suitable for investigating opinions, beliefs and perceptions on non-sensitive topics, like smoking [25]. We held separate focus groups for participants of low and high SES, because SES has shown to be an important factor in the outcome beliefs of smoking [26], and the prevalence of hard-core smokers is higher among those with a lower SES [6]. We based SES on the highest completed level of education (Dutch abbreviations in brackets), because education has shown to be a good predictor of SES in the Netherlands [27]. Low SES had primary education, lower secondary education (MAVO), or lower to middle level vocational education (LBO, MBO). High SES had higher secondary education (HAVO, VWO) or tertiary education (HBO, University). Of the 11 groups, 4 were conducted among low SES current hard-core smokers, 3 among high SES current hard-core smokers, 2 among low SES former hard-core smokers and 2 among high SES former hard-core smokers.

At the start of the interviews written informed consent and demographic data were obtained. Participants also completed the Dutch version of the Fagerström Test for Nicotine Dependence (FTND; [28,29]). Participants were ensured their responses were anonymous and would only be used for research purposes. Each focus group lasted ± 45–75 minutes and was led by a moderator skilled in qualitative methods. To avoid biased responses, we selected moderators who had little prior experience with research on tobacco control (BS, EW).

Participants were first asked what they personally consider to be important pros and cons of smoking. They then completed a questionnaire (± 5 minutes) in which they listed what they personally consider to be the three most important pros and cons of smoking. We used the same procedure (i.e., first a group discussion, then the questionnaire) to assess the pros and cons of quitting. At the end of the discussion, we probed for additional reasons and arguments to smoke or to quit smoking.

The study protocol was approved by the Medical Research Ethics Committee of the Erasmus MC.

### Analysis of the structured questionnaires

The questionnaire data were analysed in three steps. First, we imported the data in QSR NVivo 8 and we coded all perceived pros and cons of smoking and quitting listed in the questionnaire data *in vivo*. These questionnaire data yielded 145 separate codes (i.e., 145 separate pros and cons of smoking and quitting).

In the second step, two authors (JB, MS) independently extracted main categories based on the thematic content of the codes. Consensus among the coders was high. They then met to refine the main categories and to distinguish (when necessary) subcategories. Together they arranged all survey codes among different categories, reaching full consensus.

In a third step we quantified all codes which were classified in the previous stage. Since no participant listed the same pro or con twice, the number of references for each code also represented the number of participants who explicitly reported this specific pro or con. This allowed us to compare different pros and cons, using these numbers of participants. It also allowed us to compare categories based on the portion of all references within a perspective (i.e., pros of smoking, cons of smoking, pros of quitting, cons of quitting). Finally, we compared the four different perspectives based on the content of their categories In Table 2, we present these data according to smoking status, but not according to SES group, because we found no relevant differences there.

**Table 2:** Perceived pros and cons of smoking and quitting

	**Smoking**				**Quitting**			
	***Pros***		***Cons***		***Pros***		***Cons***	
	**Current HCS**	**Former HCS**	**Current HCS**	**Former HCS**	**Current HCS**	**Former HCS**	**Current HCS**	**Former HCS**
1. **Finance**	**∙**		c ^a^	c	p ^a^	p	**∙**	
2. **Health**	**∙**		cc	cc	pp	pp	**∙**	**∙**
Serious problems and diseases			**∙**	**∙**	**∙**		**∙**	**∙**
Minor problems	**∙**		**∙**	c	**∙**	p	**∙**	**∙**
Physical fitness			c	c	p	p	**∙**	
Hygiene			**∙**	**∙**	**∙**	**∙**		
General health					p	**∙**		
Appearance			c	**∙**		**∙**		**∙**
3. **Intrapersonal Processes**	ppp	ppp	**∙**	c	p	p	ccc	cc
Addiction/Dependence	p	p	**∙**	c			cc	c
Stress	pp	pp					c	c
Adherence to rules	**∙**		**∙**		**∙**	**∙**		
KillingTime	**∙**	**∙**		**∙**		**∙**	**∙**	**∙**
*Miscellaneous*	**∙**	**∙**			**∙**	**∙**		
4. **Social Environment**	p	pp	c	c	**∙**	**∙**	**∙**	c
Children			**∙**	**∙**	**∙**			
Social Exclusion	p	pp	c	**∙**	**∙**	**∙**	**∙**	c
*Miscellaneous*		**∙**	**∙**	**∙**	**∙**	**∙**		**∙**
5. **Physical Environment**			c	c	**∙**	p		**∙**
Odours			c	c	**∙**	p		**∙**
Safety			**∙**	**∙**	**∙**	**∙**		
6. **Food and Weight**	**∙**	**∙**		**∙**	**∙**	p	c	c
*Other*	**∙**				**∙**		**∙**	
*No Pros or Cons*	**∙**	**∙**	**∙**	**∙**	**∙**	**∙**	**∙**	c

^a^The perceived pros and cons for current and former hard-core smokers. The number of p’s (c’s) represent the percentage of all pros (cons) within a subgroup: (< 10%), p or c (10–30%), pp or cc (30–50%), ppp or ccc (> 50%). Themes in bold represent main themes.

### Analysis of the transcripts

The transcript data were also analysed in three steps. First, we conducted a procedure for note based analysis [25]. In this technique, the co-moderator makes notes during the focus group to capture important non-verbal behaviours of the participants. The leading moderator listens for inconsistent, vague or cryptic comments and probes for understanding. For each issue the moderator offers a summary of the answers to key questions and seeks confirmation from the focus group participants*.* Immediately after the interview, the moderator and co-moderator debrief and note additional themes, hunches, interpretations and ideas. The recordings of the focus groups are then transcribed verbatim (JB).

In a second step two authors (JB, MS) independently coded the interview transcripts of one focus group for thematic content. Consensus was high and, after discussing the codes, the authors reached full consensus over the coding procedures. The first author (JB) coded the remaining transcripts accordingly, which yielded 188 separate codes. The coding of these remaining transcripts was overseen by two other authors (TS, MK) to ensure reliability.

In the third and final step, two coders (JB, MS) arranged the codes from the focus group transcripts into the main and subcategories found in the questionnaires. We used the same classification as used for the questionnaires, because the questionnaires served as summary for the participants: i.e. participants listed their most important pros and cons immediately after discussing them. Some pros or cons could be categorized in more than one theme, but for matters of clarity, we categorized every single pro and con in just one single (sub)category. The initial difference in coding between the two authors was acceptable with 85.1% agreement consensus on main categories. Full consensus (100%) was easy to reach. Percentage agreement is a commonly used method of calculating intercoder reliability [30]. Together, the two coders evaluated the main categories and the subcategories once more to ensure the validity of these categories.

## Results

### Main categories

We used the qualitative data of the questionnaires and the transcripts to group the perceived pros and cons of smoking and smoking cessation into 6 main categories and 14 subcategories. For each main category we selected one exemplary quotation that best reflects that main category. These quotations are presented in Table 3.

**Table 3:** Example quotations

**Main category**	**Example quotation**	**Participant**
1. Finance	That is what made me quit smoking. It costs too much money. And at that time I did not have a lot of money, but I smoked it all away, until I thought: “What am I doing?”	Female former hard-core smoker with high SES
2. Health	In those days [when I smoked], when I had a cold, I sometimes had a cough for over four, five weeks. And I always had to have a handkerchief with me. Nowadays, I have a handkerchief with me for over four days, without even using it. And when I have a cough, it is gone in two days.	Male former hard-core smoker with low SES
3. Intrapersonal Processes	A con [of quitting] was that in the beginning I felt something was missing, I did not know what to do. […] But I got rid of those cravings within a couple of months. I did not worry too long.	Male former hard-core smoker with low SES
4. Social Environment	Only smoking neighbours visit me […] partly because we are neighbours. But non-smoking neighbours do not visit us and we do not visit them. We even do not visit some relatives who do not want you to smoke in their house.	Male current hard-core smoker with low SES
5. Physical Environment	Yes, I loved it when I had quit. Everything was much fresher. […] For me, the biggest advantage was that that my house was clean and fresh.	Female former hard-core smoker with low SES
6. Food and Weight	I quit smoking twice. […] The first time I gained 13 kilos and the second time, about five years ago, I gained 24 kilos. I was so deeply unhappy. […] It was madness. I will never do it again, I will never quit smoking again.	Female current hard-core smoker with low SES

Note: Example quotation for each main category with participant information.

The first main category was ‘Finance’ and entailed the perceived financial pros and cons. Because all arguments in this category concerned the financial costs of smoking and the absence of these costs when one has quit, we did not identify any subgroups here.

The second main category we found was ‘Health’, which included physical health consequences of smoking and quitting. In this category we distinguished 6 subcategories: ‘Serious problems and diseases’, ‘Minor health issues’, ‘Physical fitness’, ‘Hygiene’, ‘General’, and ‘Appearance’. The first two subcategories included several health-related issues ranging from cancer to coughing. We considered long-term, life-threatening issues (such as cancer) to be a serious problem and more short-term, non-lethal issues (such as coughing) to be a minor health issue. When participants reported arguments about their perceived level of energy or tiredness we placed these arguments in the ‘Physical fitness’ subcategory. We categorized physical changes related to hygiene (e.g., bad breath, yellow fingers and bad teeth) in the ‘Hygiene’ subcategory and physical changes related to one’s overall appearance (e.g., unhealthy looking skin or hair) in the ‘Appearance’ subcategory. We placed more abstract remarks, like “smoking is bad for my health” and “quitting will improve my health”, in a ‘General’ subcategory. Interestingly, participants tended to focus more on short-term health consequences than on long-term health effects of smoking.

The third main category was ‘Intrapersonal Processes’. This was the most diverse and therefore least straightforward of all the main categories. However, almost all of the perceived pros and cons grouped here were related to the psychological and physiological factors caused by nicotine intake. The accompanying subcategories were: ‘Addiction and Dependence’, ‘Stress’, ‘Adherence to Rules’, and ‘Killing Time’. Although the first two subcategories show some overlap (many stress-related pros and cons could, for example, also have been categorized as arguments related to ‘Addiction and Dependence’), these two categories are still fundamentally different. In general, the ‘Addiction and Dependence’ category included the physical aspects that maintain the tobacco addiction (e.g., feelings of pleasure or reward). The ‘Stress’ subcategory, on the other hand, described the psychological aspects of the addiction and mainly includes (internal and external) triggers to smoke, like negative emotions and stress-factors. Finally, ‘Adherence to Rules’ described the (psychological) effects of smoking restrictions and ‘Killing Time’ entailed arguments about countering boredom. As shown in the Table 3, the psychological effects were sometimes less severe than expected beforehand.

The fourth main category was ‘Social Environment’ and included arguments involving (significant) others. We identified two associated subcategories: ‘Children’ and ‘Social Exclusion’. In general, this main category included arguments about the perceived influence others have on smokers, as well as the influence smokers have on others. The subcategory ‘Children’ entailed arguments about one’s own children, as well as the children of others and children in general. Arguments about the perceived level of social integration within the family, among friends or in society were placed in the ‘Social Exclusion’ subcategory.

The fifth main theme entailed the ‘Physical Environment’ of the smoker. Within this main category we identified the subcategories ‘Odours’ and ‘Safety’. Most arguments here focussed on the smell smoking causes in clothing, house or car (i.e., ‘Odours’). ‘Safety’ arguments were usually about the dangers of causing fire.

The sixth and final main category included pros and cons concerning ‘Food and Weight’. The arguments in this category were usually about the changes in body weight due to quitting and the accompanying related change in diet [31]. Due to the large homogeneity of the pros and cons, we did not distinguish any subcategories here.

Since we aimed to categorize all pros and cons, an additional category was created. The very small number of arguments which could not be grouped in any of the six categories described above were classified as “Other”. An example of such an argument comes from one smoker who reported he “liked to blow smoke rings”.

We labelled all statements from current and former hard-core smokers who said that they could not come up with any argument for one of the perspectives as “No pros or cons”. For example, a few former smokers could not remember any con of quitting, while some of the current smokers could not come up with any pro of smoking.

### Differences between perspectives

Although the pros and cons of smoking largely mirrored those of quitting, there were some noticeable differences. Many pros of smoking (e.g., feelings of pleasure) were also mentioned as a con of quitting (e.g., missing moments of pleasure). Conversely, many cons of smoking (e.g., health problems) corresponded with certain pros of quitting (e.g., better health). Although there were many similarities, four major differences emerged.

The most pronounced difference was found the Food and Weight category. Many smokers and former smokers indicated that quitting makes one gain weight (con of quitting). Conversely, almost no one reported that they smoked to lose or keep weight. Apparently, weight is only an issue for quitting, but not for smoking.

The second major difference was in the Social Environment category. Although being part of a group was usually considered an important pro of smoking, quitting does not necessarily mean that one is no longer part of that group. Therefore, social ingratiation is usually an important pro of smoking, but to a lesser extent a con of quitting.

Thirdly (although less visible in Table 2), children appeared to be a very good motivator to quit smoking, but did not serve as a prominent con of smoking. Many smokers mentioned that their second-hand smoke does not harm their children, because they do not smoke in the presence of children. Consequently, hardly anyone reported negative effects on children as a con of smoking. However, many did mention many positive effects of quitting on children (or pregnancy). If someone quits smoking, he or she is considered to be a good example for their children. Also, these children will not be exposed to second hand smoking (anymore).

Fourthly, many smokers mentioned the reduction of stress as an important motivator to smoke. However, not having this relaxant seemed less important as a con of quitting, especially for former smokers. Perhaps they had found another way of reducing feelings of stress.

## Discussion

### Overview

In this focus groups study we identified 6 main categories and 14 subcategories in perceived pros and cons of both smoking and quitting in current and former hard-core smokers. The results suggested that the four different perspectives on smoking and quitting (i.e., pros of smoking, cons of smoking, pros of quitting, and cons of quitting) are essentially different. We found few pronounced differences in perceived pros and cons between current and former smokers and no differences between participants of high and low SES.

### Main categories

Finance appeared to be an important con of smoking and pro of quitting. Smoking is relatively costly and tobacco products continue to increase in price. Many countries have implemented policies to increase the price of tobacco products and these policies are thought to target low SES smokers in particular [32,33]. In our focus group study, however, we found no indication that low SES smokers are more affected by cigarette prices than high SES smokers. Both groups reported this theme equally often.

Health was a second major con of smoking and pro of quitting. Both smokers and former smokers reported that smoking lowers one’s physical fitness and makes one less attractive (e.g., fainted skin or hair). Smoking also causes minor health problems (e.g., coughing) and is sometimes associated with bad hygiene (e.g., yellow fingers and bad teeth). Quitting is believed to negate these negative effects of smoking. It was interesting that the participants hardly mentioned major health problems like lung cancer or cardiovascular diseases. Many anti-smoking campaigns use these major health issues as their main argument [34], but hard-core smokers may be unaffected by these messages.

The third major category we distinguished was Intrapersonal Processes. Current smokers, in particular, deemed these arguments to be important pros of smoking and cons of quitting. However, participants reported pros and cons in all four perspectives, emphasising the importance and diversity of this theme. It was reported that smoking gives feelings of pleasure and relieves tensions. Nevertheless, when someone quits, he or she will temporarily miss these feelings of pleasure and may find it difficult to relieve stress. Former smokers recalled that these negative effects turned out better than expected. They did not experience withdrawal symptoms as much as the current smokers currently anticipate. Perhaps these accounts of former smokers may help convince current smokers to quit.

The social environment was also an important topic. Both current and former smokers mentioned that, in their early teens, smoking helped them make friends and made them feel part of a group. Later on in life, however, smoking lost a significant part of this social function. For former smokers, this was still an important pro of smoking. Although many current smokers mentioned that smoking still makes them feel comfortable among friends and strangers, they also reported feeling like a societal outcast due to all the tobacco control policies and smoking restrictions in the Netherlands. Smokers also mentioned receiving many negative comments about their smoking behaviour from non-smokers and former smokers, and that these people try to convince them to quit. Not surprisingly, not receiving these comments and no longer feeling a social outcast were considered pros of quitting. These results are in line with others who emphasised the influence of the social network on smoking and quitting behaviour [35].

The fifth theme was Physical Environment, which mostly contained cons of smoking and pros of quitting. The majority of arguments in this theme were about the smell and stench from smoking. Both current and former smokers reported that smoking makes their house, car, and clothes smell and acknowledge that quitting will make this smell disappear over time. Since most smokers are aware of this con of smoking, this could be a relevant topic for future research on third hand smoking (i.e., consequences of tobacco smoke that linger after the cigarette has already been extinguished). Third hand smoking has been investigated in houses [36] and cars [37] but could also be a topic in interventions targeting hard-core smokers.

The sixth theme was Food and Weight and was only found relevant as a con of quitting. The arguments in this category were about gaining weight after quitting and an (often) accompanying change in diet. Many smokers expect to gain weight after quitting, which was confirmed (but to a lesser extent) by the former smokers. This theme appears to be specific to the cons of quitting as no similar arguments were given in the other perspectives.

Finally, we found that some participants were unable to generate any pros of smoking or cons of quitting. Despite having smoked for many years, they could not give any rationale for their smoking behaviour. For some participants this was quite an eye-opener. In a clinical setting, emphasising that one does not have any pros of smoking, may serve as a starting point for some smokers to consider quitting.

### Differences between subgroups

We found few major differences in perceived pros and cons between current and former smokers. In general, former smokers seem to have a more comprehensive view on both smoking and quitting. While many current smokers tend to focus on the barriers of quitting, former smokers are usually more positive about smoking cessation. This is probably due to the change of beliefs after quitting. It is known that outcome beliefs tend to shift after quitting [38], and perhaps the longer one has quit, the larger the shift. In our study, the number of years quit ranged between 1.5 and 40 years. We were therefore able to capture the outcome beliefs from various time stages after quitting. Secondly, many former smokers did not experience major negative consequences (e.g., gaining weight or extreme withdrawal effects), or only to a slight extent. However, former smokers discovered some unexpected benefits of quitting, like regaining their taste and appetite.

We held different focus groups for low and high SES participants, because SES has shown to be an important factor in the outcome beliefs of smoking [26], and the prevalence of hard-core smokers is higher among those with a lower SES [6]. However, no notable difference emerged between the two different socioeconomic groups. We also found no difference between men and women. Even on the topic of weight control, where some found substantial differences [39], we found no indication that more women than men consider this an important con of quitting. However, this may be due to the relatively small sample size.

### Proposing the Distorted Mirror Hypothesis

Although the pros and cons of smoking and the pros and cons of quitting show a similar pattern, there are some differences. Therefore, we propose the Distorted Mirror Hypothesis. According to this new hypothesis, many pros of smoking are similar to certain cons of quitting. Conversely, many cons of smoking correspond to certain pros of quitting. Like a mirror, the pros and cons of smoking are reflected in the cons and pros of quitting, respectively. This mirror, however, is distorted: not all pros (and cons) of smoking are similarly reflected in the mirror of quitting (e.g., arguments related to social cues). Further, the mirror of quitting also reflects elements that do not exist in the pros and cons of smoking (e.g., arguments related to weight).

Four major differences were found in the ‘distorted mirror’: a) weight gain is an important con of quitting, but weight loss or maintenance are not important pros of smoking, b) social integration is an relatively important pro of smoking, but losing friends is not a con of quitting, c) saving the health of children is a pro of quitting, but harming these children with smoke is not a con of smoking, and d) stress reduction is an important pro of smoking, but this seems less important as a con of quitting.

This knowledge could be useful in future research or interventions targeting (hard-core) smokers. A clinician treating smokers who consider social integration as an important pro of smoking, could point out that quitting is not likely to isolate these smokers from their social environment. Tobacco control advertisements targeting hard-core smokers are advised not to focus on the possible harms of smoke to children, but to focus on the health benefits of quitting for these children. Similarly, interventions targeting smokers who use tobacco as a way to relax may use the accounts of former smokers to inform current smokers that it is possible to find relaxation after quitting. The differences brought forth by the distorted mirror may help to increase the effectiveness of interventions targeting these specific cognitions. The importance of message framing has been emphasised before [40]. Framing a health message as a gain has different effects on persuasion than framing the message as a loss. Research on the framing of smoking cessation messages has further shown that these effects are influenced by gender and health risk perception [41]. Different groups of (hard-core) smokers need different messages. Therefore, it is important that this distinction is also made clear in future research on the perceived pros and cons of smoking and quitting.

### Study limitations

Our study may be limited in the extent to which the results are generalizable. Considering our relative small sample size, the results are not statistically generalizable. However, the aim of our study was to uncover all possible pros and cons within the population of hard-core smokers and to generalize to broader concepts and theory. Our results are therefore what Polit and Beck [42] described as analytic generalizable: using individual qualitative data to find broader constructs or theory that are applicable to the entire (sub)population. We therefore believe that the pros and cons we have found and the theory we formulated, are applicable to the Dutch population of hard-core smokers as a whole. Future quantitative research may investigate the statistical generalizability of these pros and cons. Also, the causal relationship between these pros and cons and actual smoking or quitting behaviour could not be determined by the current qualitative research and future quantitative research may provide more insight in this as well.

Some pros or cons may have been left unmentioned by participants, because of the group setting in which the interviews were carried out. For example, concerns about sexual activity have not been expressed, perhaps because participants did not feel comfortable sharing those. Also, there are topics (e.g., partners) that are not cited in this paper. These topics may have been implied in more general remarks about social environments, but were never mentioned explicitly.

In our study we used the reports of former hard-core smokers to gain a more comprehensive view on the pros and cons in current hard-core smokers. These reports must be interpreted in the light of former smokers’ current smoking status. Smoking-related cognitions tend to change after quitting [38], and the narratives of the former smokers may therefore be influenced by retrospective recall. However, almost all pros and cons mentioned by current smokers were also mentioned by former smokers, and vice versa. Since the main aim of our study was to gain knowledge on hard-core smokers in general (not only the former smokers), the influence of retrospective recall on our general results is limited.

Another possible limitation of our study could be our definition of a hard-core smoker. Although various definitions are applied in this field [2], we used three well-known core concepts: relative high tobacco consumption, little intention to quit and a resilience to societal pressures. The most notable difference between our definition and that of others, is that we only included smokers who have smoked more than 15 years in their lifetime. Many studies acknowledge the resilience to societal pressures to quit as a characteristic of ‘hard-coreness’, but set a less tight criterion (i.e., included smokers who smoked daily for only the past five years). On the other hand, recent research also suggest that the number of years smoked does not influence the effectiveness of quitting attempts [43]. Consequently, the differences between the studies of others and ours related to the number of years smoked is probably negligible.

A recommendation for future research is to incorporate other factors that could play a role in predicting different pros and cons. In our study we compared participants based on their smoking status (current vs. former smokers), their SES (low vs. high) and, to a lesser extent, gender. However, other predictors of pros and cons may also play a role in this respect. Nicotine dependence, for example, may change one’s attitudes towards smoking and quitting. Similarly, these attitudes may be influenced by personality traits, self-efficacy, features of the social environment and demographic characteristics (e.g., occupation, age, having children). It is established that smoking behavior (and therefore cognitions about smoking and quitting) differs across countries [44]. Since we only included Dutch hard-core smokers, country of origin may also have been a potential biasing factor. These topics were beyond the scope of this study, but future research may investigate the relation between the perceived pros and cons and these variables more thoroughly.

## Conclusions

In this study we categorized the perceived pros and cons of smoking and quitting into 6 main categories: Finance, Health, Intrapersonal Processes, Social Environment, Physical Environment, and Food and Weight. Although the perceived pros and cons of smoking in hard-core smokers largely mirror the perceived pros and cons of quitting, major differences should be taken into account that can be addressed in interventions motivating hard-core smokers to quit. With the Distorted Mirror Hypothesis, this paper therefore addresses an important deficit in our understanding of the pros and cons of smoking. This paper also advances the currently limited literature on hard-core smokers. Future research may address both topics more thoroughly.

## Abbreviations

SES: Socioeconomic status; FTND: Fagerström Test for Nicotine Dependence.

## Competing interests

The authors declare that they have no competing interests.

## Authors’ contribution

TS, MK and DM wrote the original proposal for this study. JB, BvS and EW conducted the focus group interviews. JB and MK coded all transcripts and questionnaires, and this was reviewed by TS and MK. All authors contributed to and approved the final version of this manuscript.

## Pre-publication history

The pre-publication history for this paper can be accessed here:

http://www.biomedcentral.com/1471-2458/14/175/prepub
